# Effect of *Ruta graveolens* and *Cannabis sativa* alcoholic extract on spermatogenesis in the adult wistar male rats

**DOI:** 10.4103/0970-1591.33720

**Published:** 2007

**Authors:** M. R. Sailani, H. Moeini

**Affiliations:** Department of Biology, Faculty of Sciences, Isfahan University, Isfahan, Iran; * Department of Biology, Faculty of Sciences, Shahid Chamran University, Ahvaz, Iran

**Keywords:** *Cannabis sativa*, fertility-decreasing properties, *Ruta graveolens*, spermatogenesis

## Abstract

**Objective:**

The present study was undertaken to evaluate the effects of alcohol extracts of *Ruta graveolens* and *Cannabis sativa* that were used traditionally in medieval Persian medicine as male contraceptive drugs, on spermatogenesis in the adult male rats.

**Materials and Methods:**

Ethanol extracts of these plants were obtained by the maceration method. The male rats were injected intraperitionaly with *C. sativa* and *R. graveolens* 5% ethanol extracts at dose of 20 mg/day for 20 consecutive days, respectively. Twenty-four hours after the last treatment, testicular function was assessed by epididymal sperm count.

**Result:**

The statistical results showed that the ethanol extracts of these plants reduced the number of sperms significantly (*P*=0.00) in the treatment groups in comparison to the control group. The results also showed that the group, treated by extract of *R. graveolens* reduced spermatogenesis more than the group treated by extracts of *C. sativa*.

**Conclusion:**

The present study demonstrated the spermatogenesis reducing properties of the ethanol extracts of *R. graveolens* and *C. sativa* in the adult male wistar rats but more studies are necessary to reveal the mechanism of action that is involved in spermatogenesis.

## INTRODUCTION

One of the important concerns today is the problem of overpopulation. The population of the world is growing faster than the supplies of food, shelter and fuel. Therefore finding safe and effective contraceptive materials can be useful for this aim. In addition, many individuals throughout the world prefer to use natural remedies as a treatment option.

The plant therapy's literature is related to that of humanity, because in most cultures man has always depended on the curative values of medicinal herbs to cure some illnesses. In some cultures, namely in Persia and India as well as in Europe and North America, the plant therapy is more and more appreciated especially for its holistic approach.

Previous studies have shown the aphrodisiac, anti-fertility effects and fertility-enhancing properties of some plant extracts. *Acasia farnesiana*, *Anacyclus pyrethrum*, *Aframomus melegueta*, *Piper guieneense* and *Lepidium meyenii* have aphrodisiac effects.[1–5] The root of *Lepidium meyenii* (Maca) is used for its fertility-enhancing properties[1] or *Camphore* (*Cinnamomum camphora*) is traditionally used as an abortifacient, contraceptive and antiaphrodisiac.[6] The fertility reducing effects of *Ruta graveolens*,[7] Gossypol (an extract of cottonseed),[8] *Chenopodium ambrosioides L.*[3] have been studied. Moreover, in Medieval Persian medicine, the plants such as *Gossypium herbaceum, Cyperus longus and Vitex psedonegundo* have been considered as fertility-decreasing agents.[6] In Medieval Persian Medicine, physicians categorized medicinal herbs from Africa to china and studied them in a scientific manner. These practitioners had brought great contributions to pharmaceutical science. The unfertilization effects of *Ruta graveolens* and *Cannabis sativa* (Plants selected from the medieval medical texts of Persia such as Qanon - fel Teb (The canon) by Ebn-e-sina (980-1037)) were investigated in this study. In recent years, some experimental studies have evaluated medieval Persian natural therapies using modern scientific methods. Therefore we considered in our study, the effects of *Ruta graveolens* (*Rue*) and *Cannabis sativa* *L*. extracts on Sperm production. *Rue*, a hardy, evergreen, somewhat shrubby plant, is native to southern Europe and northern Africa. *Rue* has emmenagogue, ecbolic, anthelmntic and antispasmodic properties.[9] Infusions and decoctions of aerial parts of Rue are used as anti-inflammatory and anti-rheumatic medicine and for the treatment of hypertension, skin illness and rhinitis.[9] *Cannabis sativa* L; Common historical uses include carminative, astringent, aphrodisiac, antiemetic and anti-inflammatory.[6,9]

## MATERIALS AND METHODS

### Animals

Adult male wistar rats weighing 250-300 g were used in this study. The rats were purchased from the animal house of Razi research center (Tehran-Iran), The rats were housed in a clean room at 25±3°C and 55±5% humidity for 3 days, before the experiments. The rats were fed a standard laboratory food (see below).

### Extraction method

The seeds of *C. sativa* and leaf of *R. graveolens* were collected from Iranian plants species and dried in shade (25°C) by the air drying method for 7 days and then, were grinded with electrical grinder. The extracts of them were obtained by the maceration method with 80% ethanol in 300 gr/Lit for 48 h.

The extracts were concentrated by Rotary Evaporator (Büchi, Water bath B-480, Switzerland) with a vacuum pump and were dehydrated in desiccators with vacuum pump. Dried extracts were resuspended in ethanol - dH_2_O (1:20) at dose of 20 mg/mL.

### Experimental design

The wistar male rats were divided at random into 3 groups of 10 animals each. All of them had the same weight, race, age and sexuality. Group 1 received 1 ml/day of 5% ethanol extract of *C. sativa* (20 mg/day). Group 2 received 1 mL/day of 5% ethanol extract of *R. graveolens* (20 mg/day). Group 3 as control, received 1 ml/day of 5% ethanol. Each group was kept in chamber (1.5 m^3^) at 25±3 ° C and 55±5% humidity for 12 h at light and 12 h at dark illumination schedule. The diet was standard rat chow containing 1.03% calcium, 0.70% phosphorus and 200 IU vitamins D_3_/100g (MF oriental Yeast Co. Ltd., Tokyo, Japan). All animals were allowed free access to food and water. All the experiments were performed between 8 to 13 o'clock.

### Epididymal sperm count

24 hours after cessation of treatment, the rats were sacrificed. Their testes and epididymides were removed and each epididymis was minced and placed in to a Warring blender (Polytron, Kinematica, Littau/Luzern, Switzerland) containing 75 ml of dissociation solution (normal saline with sodium azide [0.25%] and Triton X-100 [0.05%]) and homogenized for 2 min at the fastest speed (setting 6). The mixture was then allowed to settle for 1 min to enable the foam to dissipate. After gentle swirling to resuspend the sperms, a 1-mL sample was taken and stained by adding two drops of eosin solution (1% eosin Y in water) followed by incubation for 45-60 min at room temperature.[8,10,11] Sperm numbers were counted using a hemocytometer. Data are referred as 10^8^ sperms per epididymis.

### Statistical analyses

The data were analyzed using the Statistical Package of Social Science (SPSS) Software under Windows 14.5^th^ version (SPSS Inc, Chicago IL). All the reported values were expressed as means ± Standard Deviations (SD). Error Bar Graph (Mean ± Standard Error of Mean) was used too. The data were also analyzed using One Way ANOVA fallowed by Duncan Multiple's Range Test (DMRT) among the three groups (*P* < 0.05).

## RESULTS

### Sperm counts

**Figure 1:**
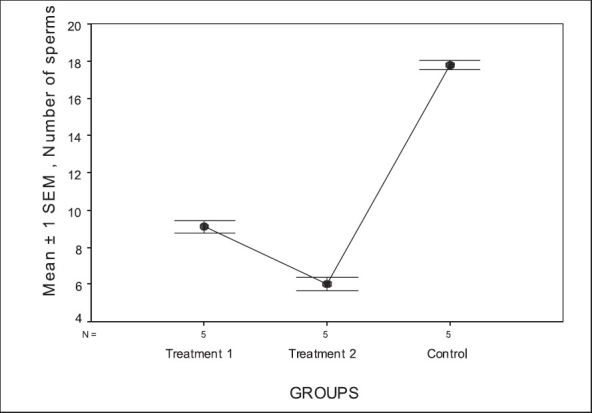
Effect of *C. sativa* and *R. graveolens* extracts on sperm counts in the treated groups (group 1 and 2, respectively). Error bar graphs with mean ± 1 standard error of mean (SEM) Y-axis: Mean ± 1 SEM of number of sperms in the treatments and the control groups. The data are referred as 10^8^ sperm per epididymis.

Intraperitional injection of the extracts of *C. sativa* and *R. graveolens* to the groups 1 and 2, respectively were performed in accompanying the control group, which was similar to the treatment groups. The only difference was that the control group received intraperitional injection of the 5% ethanol instead of the 5% ethanol extracts of these plants. We used One Way ANOVA analysis for the number of sperms among the groups. There was a significant difference between the number of sperms of the treatment groups in compare to the control group (*P*= 0.00). The data were then analyzed by Duncan's multiple range test (DMRT). Epididymal sperm count was significantly (*P*=0.00) reduced in the male rats treated by the ethanol extracts of *C. sativa* and, treated by the ethanol extract of *R. graveolens* as compare to the control [Figure 1 and Table 1]. Effects of the ethanol extracts of *R. graveolens* and *C. sativa* on sperm count were shown more reduction in group 2 than in group 1 (*P*=0.009).

**Table 1:** Epididymal sperm counts in the treatment groups and the control groups.

Adult male rats	Numbers of repeat	Sperm count
Group 1	10	9.10 ± 0.74*
Group 2	10	6.00 ± 0.79***
Control	10	17.80 ± 0.57

Values are expressed as Mean±Standard deviations (SD) of 10 rats and were analyzed using One Way ANOVA followed by Duncan's multiple range test (DMRT).

* Difference from the control, *P=0.00*;

** Difference from the group 1, *P=0.009*. Group 1: The adult male rats treated with the extract of *C. sativa.*

Group 2: The adult male rats treated with the extract of *R. graveolens.*

Control: The adult male rats treated with 5% ethanol. The data are referred as 108 sperms per epididymis

## DISCUSSION

This study demonstrated the spermatogenesis-reducing properties of *R. graveolens* and *C. sativa* extracts in adult male rats. For centuries, in medieval Persian medicine, the plants such as *Gossypium herbaceum, Cyperus longus and Vitex psedonegundo*, etc. which are presented in Table 2, have been used as fertility-decreasing agents.[8,12] Recent researches have shown the fertility reducing effects of Gossypol (an extract of cottonseed), [13] *Chenopodium ambrosioides* L,[12] etc. Gossypol, an extract of cottonseed, was evaluated in China as a male contraceptive but was abandoned due to the user's problem with hypokalemia. When Gossypol was reevaluated in a multinational study, hypokalemia was not a problem. Of 134 men treated, 65% had sperm counts less than 1 million/mL and about half of men followed more than 1 year after treatment recovered to a normal sperm count.[13] Prakash *et al.* showed that the extracts of *Codonospis ovata Benth*, *Puararia tuberosa* DC, *Punica granatum* L and *Rubus ellipiticus* inhibit pregnancy in 70-90% of rats.[14]

**Table 2:** Evaluation of some plants prescribed by medieval Persia as natural male contraceptive remedies using modern scientific researches

Family	Scientific name	Part of use**	Male contraceptive properties, Refs
*Rutaceae*	*Ruta graveolens (Rue)*	Leaf, stem and root	Known*,[15,20]
*Cannabinaceae*	*Cannabis sativa (Hemp)*	Seed, leaf and stem	Known*,[18]
*Malvaceae*	*Gossypium herbaceum*	Leaf and stem	Known,[8]
*Cyperaceae*	*Cyperus longus* L	Leaf and stem	Unknown
*Verbeanaceae*	*Vitex psedonegundo*	Leaf and stem	Unknown
*Chenopodiaceae*	*Chenopodium ambrosioides*	Leaf and stem	Known,[3,12]
*Aristolochiaceae*	*Aristolocuia indica*	Stem	Known,[21]
*Punicaceae*	*Punica grantum*	Leaf and stem	Known,[14]
*Ascelpiadaceae*	*Sarcostemma acidum*	Leaf and stem	Known,[11]

* The plants that were investigated in the present study,

** The oral administration of these plants was prescribed

The reproducibility of all the data in this study was assured by the use of four repeated experiments. Antifertility properties of Rue in female rats have been previously demonstrated. For example, Kong *et al.* showed that the chloroform extracts of the root, stem and leaf of Rue have significant antifertility activity in female rats when administered intragastrically on day's 1–10 post-coitum. They isolated Chalepesin as the active component which acts at the early stages of pregnancy.[15]

As can be seen from the present study, using the ethanol extracts of Rue and *Cannabis sativa* leads to a significant reduction in the epididymal sperm counts in the treated male rats [Table 1 and Figure 1] compared to that of the control group. As shown in Table 2 sperm reduction in the treated group with the ethanol extract of *R. graveolens* is more than the treated group with the ethanol extract of *C. sativa*. These plants were used in medieval Persian medicine for a long time as male contraceptive plant drugs, therefore it seems that these plant extracts do not have important side effects on our health, but any new laboratories findings must be thoroughly evaluated and carefully implemented to avoid temporary or long term negative impacts on human healthy. The main components of *C. sativa* are cannabinoids which are ligands of cannabinoids receptors. The receptors distributed extensively in many tissues. the cannabinoid receptor type one (CB1) has been localized to ovary, uterine endometrium, testis, vas deferens, urinary bladder and other peripheral endocrine and neurological tissues.[16] It has been shown that cannabis causes low birth weight and prematurity,[17] however the presence of cannabinoid receptors in sperm,[18] guides us to propose the possibility of a natural role for Cannabis in modulating sperm function during fertilization. Rutaceae genius has been shown to have contrary effects on fertility. For instance Qarawi *et al.* have shown that *R. chalepensis* is a fertility promoting agent while Kone *et al.* and Khouri *et al.* have shown the antifertility properties of *R. graveolens*.[15,19,20] This differential effect has been previously reported with species and extraction method.[19] The present investigation clearly shows that the ethanol extract of *R. graveolens* promoted a decreased male wistar rat sperms. It could be inferred that the treatment may act directly or indirectly on the pituitary gland secretary function causing to a decrease in the androgen. It has been demonstrated that the process of spermatogenesis and the accessory reproductive organs functions are androgen dependent.[20] Therefore, any changes in the androgen production would reflect and explain the decrease in the number of sperms.

## CONCLUSION

As a conclusion, *R. graveolens* and *C. sativa* alcohol extracts can be suggested as agents against male fertility. Maybe there are materials in these plant extracts which lead to a reduction in the number of sperms. The question that remains to be answered is, how do these plant extracts reduce number of sperms immensely? Effects of these plant extracts on sperm motility and testis histological studies will be very helpful to understand the mechanisms of action involved in these plant compounds.
